# Marine Actinomycetes with Probiotic Potential and Bioactivity against Multidrug-resistant Bacteria

**DOI:** 10.22088/IJMCM.BUMS.7.1.44

**Published:** 2018-04-03

**Authors:** Hamed Norouzi, Abolghasem Danesh, Mojtaba Mohseni, Mohammad Rabbani Khorasgani

**Affiliations:** 1 *Department of Biology, University of Isfahan, Isfahan, Iran.*; 2 *Biotechnology Research Center, Pharmaceutical Technology Institute, Mashhad University of Medical Sciences, Mashhad, Iran.*; 3 *Department of Microbiology, University of Mazandaran, Babolsar, Iran.*

**Keywords:** Marine actinomycetes, antimicrobial activity, Caspian Sea, multi-drug resistant bacteria, anti-vibrio

## Abstract

Considering antimicrobial resistance problem, marine microorganisms with the bioactivity against multi-drug resistant (MDR) pathogens have attracted many scientific interests. To address this issue, a total of 21 marine actinomycetes isolated from the Caspian Sea have been screened out. Primary screening via cross-streak method revealed that 3 strains: MN2, MN39, and MN40 produce antimicrobial agents with wide spectrum activity. In the second step, the potent strains were characterized morphologically, and then identified genetically using 16S rRNA analysis. After that, the bioactivity of the ethyl acetate extracts of liquid culture against some MDR bacteria has been studied using disc diffusion method. Finally, the exoenzymatic activity of the strains, and the anti-vibrio activity of the extracts have been evaluated. The nucleotide sequence of the 16S rRNA gene (1.5 kb) showed that the potent strains belong to the genus *Streptomyces*. The results of disk diffusion method indicated that among the 3 potent isolates, MN39 and MN2 produce biomolecules with antibacterial activity against MDR bacteria specially methicillin-resistant *Staphylococcus aureus *(MRSA) and vancomycin-resistant *Enterococcus* (VRE). In addition, potent strains showed remarkable anti-vibrio activity as well as extracellular enzyme production including amylase and protease. The results of this study revealed that the marine actinomycetes isolated from the sediments of Caspian Sea produce biomolecules effective against MDR bacteria, and suggested that these strains deserve to be studied as potential probiotics due to their anti-vibrio activity besides exoenzyme production.

The appearance of antimicrobial resistance against common drugs has become a serious global problem in healthcare. Discovery of novel drugs active against multidrug-resistant (MDR) bacteria is a main area of researches which try to combat antimicrobial resistance threat (1). Natural products are infinite sources of bioactive molecules with useful biological activities (2, 3). Marine environment which covers almost 70% of the earth surface, contains a lot of microorganisms capable of producing bioactive agents (4, 5). Marine Actinobacteria are Gram-positive and filamentous bacteria which are recognized as important secondary metabolite producers (6). Among the Actinobacteria, genus *Streptomyces* is a powerful producer of functional metabolites with broad range of pharmaceutical activities such as antimicrobial, antitumor, antiviral, and probiotic activity (4, 7). In addition, it is apparent that the marine environment could be a source for finding new Actinobacteria with novel bioactive metabolites or biologically active compounds that may be used as biocontrol agents (8, 9). Today, there are some studies that have considered marine actinobacteria as potential probiotic in aquaculture since these marine strains are able to inhibit the growth of *Vibrio spp*. (8, 10). The present study began by screening marine sediments of Caspian Sea for isolating Actinobacteria with antimicrobial activity against MDR bacteria, and then the ability of selected strains to produce exoenzymes as well as their anti-vibrio activity has been investigated.

## Materials and methods

**Microorganisms**

The 21 actinomycete strains used in this work had been isolated before from Caspian Sea sediment (11) and deposited to the microbial laboratory of school of Pharmacy, Mashhad University of Medical Sciences, Mashhad, Iran. Viability of all strains was maintained on Starch Casein Agar (SCA) slants at 4 °C. For longer storage, each strain was grown on nutrient broth at 28 °C. After 7 days, glycerol was added to the final concentration of 15% and stored at -20 °C (11).

**Indicator microorganisms**

The MDR strains of *Salmonella typhi *ATCC 3311, methicillin-resistant *Staphylococcus aureus* ATCC 33591, *Pseudomonas aeruginosa* ATCC 2108, *Acinetobacter* *baumannii* ATCC 1605, *Escherichia coli* ATCC 2452 and *Enterococcus faecium* ATCC 700221 were used as indicator microorganisms which were maintained in glycerol stock at -20 °C. All the indicators have been revived via inoculating on nutrient agar (NA) followed by incubating at 37 °C for 24 h.

**Primary antibacterial screening using cross streak method**

Isolates were screened out against Gram-negative and Gram-positive MDR bacteria using cross streak method. The actinomycete isolates were inoculated in straight line on NA plates, and incubated at 28 °C for 3 days. Fresh sub-cultured MDR strains were prepared in nutrient broth until the visible turbidity became equal to that of 0.5 McFarland and then, streaked perpendicular to the plates on which actinomycete strains had been grown previously. After incubating at 37 °C for 24 h, the microbial inhibition was evaluated by determining the diameter of the inhibition zones (5, 12). The isolates that showed production of bioactive compounds which were active against all MDR indicators, have been selected as potent strains for further studies.

**Characterization of potent strains**

The morphological and physiological characteristics of potent strains have been determined via culturing different media including SCA, Extract Malt Extract Agar (ISP2), Kuster's Agar medium.. After 7 days of incubation at 30 °C, their morphological properties including absence or presence of aerial mycelia, colony reverse color, and spore-bearing hyphae were studied (13).

**DNA extraction and 16S rRNA sequencing**

Molecular identification of the potent isolates has been performed by extracting the genomic DNA using a standard bead beating method (14). After analyzing qualitatively and quantitatively via a spectrophotometer, the extracted DNA has been separated via running an electrophoresis on a 0.8% (w/v) agarose gel. The 16S rDNA gene was then PCR amplified using universal bacterial 16S rDNA primers (PA 5′-AGAGTTTGATCCTGGCTCAG-3′ and PH 5′-AAGGAGGTGATCCAGCCGCA-3′) (15). The thermocycler was programmed to denature the molecule at 95 °C for the first 5 min followed by 35 cycles of 1 min at 95 °C, 1 min at 50 °C, and 2 min at 72 °C with a final extension step for 5 min at 72 °C. The PCR products were purified using a GeneJet PCR purification kit (Thermo Scientific, Lithuania) according to the manufacturer’s instructions. Purified products were sequenced by GATC Biotech (Germany).

**Phylogenetic analysis**

For phylogenetic evaluation, all reference sequences were obtained from the National Center for Biotechnology Information (NCBI). The BLAST program (www.ncbi.nlm.nih.gov/blst) was employed in order to assess the degree of DNA similarity. Then, the sequences were aligned using the multiple sequence alignment program ClustalX. Neighbor joining phylogenetic trees were constructed using the Molecular Evolutionary Genetics Analysis package, MEGA5 (16).

**Sub-merged fermentation**

A single colony of selected actinomycetes was inoculated into 50 ml of seed medium (modified A1BFe + C) (17): starch 10 g L^−1^, yeast extract 4 g L^−1^, peptone2 g L^−1^, KBr 0.1 g L^−1^, Fe_2_(SO_4_)_3_·4H_2_O 0.04 g L^−1^, CaCO_3_ 1 g L^−1^, and sea salt 30 g L^−1^, pH 7.0, adjusted before sterilization , in a 250 ml Erlenmeyer flask. The inoculated culture was incubated at 28 °C for 54 h in a rotary shaker incubator with a speed of 180 rpm, and used as a seed stock. Then, 6 ml of the seed culture was inoculated into 100 ml of production culture which contained the same medium (modified A1BFe + C), and was shaking-incubated at 28 °C, 180 rpm. After 94 h, the cultures were Whatman-paper filtered, and centrifuged at 6000 × g for 10 min (Sigma). The cell-free supernatant was extracted three times with the equal volume of ethyl acetate, followed by concentrating the extracts via evaporating the solvent to yield a crude residue. After that, the extracts were reconstituted in 1ml ethyl acetate, and their antibacterial activity against mentioned MDR bacteria has been investigated (18).

**Secondary screening of antimicrobial activity**

**using disk diffusion method**

The antimicrobial activities of the concent-rated extracts were tested against different indicator microorganisms using agar disc diffusion method as described by Kirby-Bauer with some modifications (5). Briefly, the fresh Muller-Hinton broth (Merck) was inoculated by MDR bacteria and shaking-incubated at 37 °C at 100 rpm, overnight. When the turbidity of the indicators reached to a final cell density of approximately 10^8^ cfu ml^-1^, a swab of culture was spread as uniformly as possible throughout the entire medium. The paper discs (6 mm diameter, HiMedia, India) which had been previously impregnated with the extracts were introduced on the agar plate, and incubated at 37 °C. After 24 h, the antibacterial activity was evaluated by measuring the diameter of translucent inhibition zones formed around the discs. Paper discs (6 mm diameter, Padtan Teb, Iran) impregnated with ampicillin (10 µg/disc), gentamicin (10 µg/disc), tetracycline (30 µg/disc), cefazolin (30 µg/disc), and penicillin (10 µg/disc) were used as positive controls whereas a disc containing only ethyl acetate (30 µl/disc) was adopted as a negative control.

**Exoenzymatic assay**

Cultured actinomycetes were screened for hydrolytic exoenzymatic activities (amylase, and protease) by growing the strains on ISP 2 plates. To evaluate the amylolytic activity, starch had been added to the medium, while in order to determine the presence of protease, skimmed milk had been mixed with the content of ISP 2 medium. After incubating at 37 ºC for 24 h, the plates were flooded with 1% iodine solution, and then the presence of the amylase was visualized by forming a decolorized halo around the bacterial growth due to the digestion of starch. Proteolytic activity was confirmed when a zone of digested milk appeared around the colonies formed (8).

**Bioactivity against** ***Vibrio sp.***

The ability of potent strains to produce bioactive

compounds with anti-vibrio activity in aquaculture has been investigated using a modified cross-streak method. At first, the three potent strains were streaked on NA plates and incubated at 28 °C for 3 days. Three fish-pathogenic *Vibrio* strains (*V. harveyi*, *V. parahaemolyticus*, and *V. proteolyticus*) which had been cultured on Tryptone Soya Broth (TSB) for 18 h, were streaked on NA plates perpendicular to the grown actinomycetes (8, 10). The plates were incubated at 30 °C for another 24 h. The width of inhibition zones of each *Vibrio* species was measured in mm. In addition, to confirm the anti-vibrio activity of the producer strains, the ethyl acetate extracts obtained from liquid cultures were tested using disc diffusion method.

## Results

**Actinomycetes isolation**

**Fig. 1:**
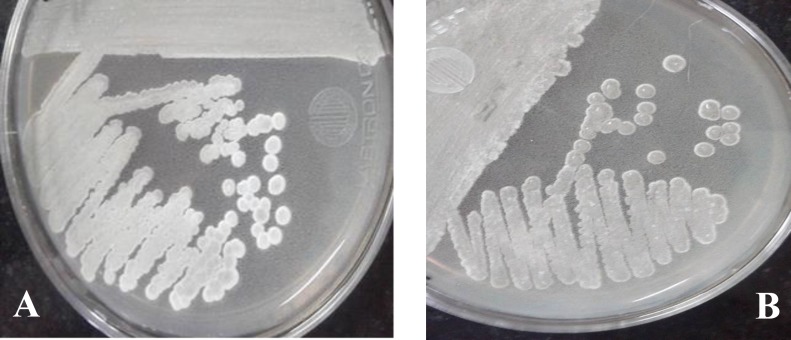
Morphological appearance of two actinomycete isolates grown on Starch Casein Agar. **A:** MN2 isolate; **B**: MN40 isolate. The colonies possess a powdery texture with branching filaments and aerial mycelia

Figure 1 shows the growth of MN2 and MN40 strains on SCA. Based on the grwoth of strains, SCA was observed to be the best medium for isolatiing and storing the actinomycete strains. It should be emphasized that all the strains formed colonies with a tough or powdery texture (Figure 1) when they grew on SCA, ISP2, and Kuster's Agar media.

**Primary antibacterial screening**

**Fig. 2:**
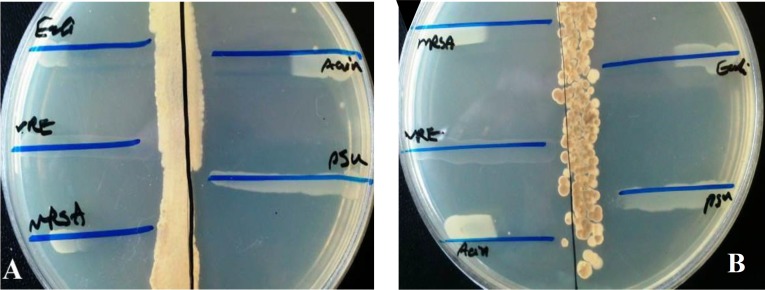
Preliminary screening of actinomycetes for antimicrobial activity using cross-streak method. Bioactive compound(s) produced by (**A**) isolate MN2 and (**B**) isolate MN40, inhibited the growth of resistant bacteria including *Escherichia coli*, Vancomycin-resistant *Enterococcus*, Methicillin-resistant *Staphylococcus aureus*, *Pseudomonas aeruginosa*, and *Acinetobacter* *baumannii* which have been grown perpendicularly to the actinomycetes MN2 and MN40

Among the 21 actinomycetes isolated from the sediments of Caspian Sea, 14 strains showed antibacterial activities against at least one of the MDR indicator bacteria (Figure 2) which has been stated in table 1. The results demonstrated that the isolated actinomycetes produce bioactive compound(s) which can inhibit the growth of MRSA, VRE and* P. Aeruginosa* more than other MDR pathogen bacteria. However, three antagonistic actinomycetes including MN2, MN39, and MN40 exhibited broad spectrum activities against all MDR bacteria (Table 1)

**M** **orphological and physiological characteristics** **of three potent strains**

Out of 21 isolates, three of them that revealed pronounced antibacterial activity were selected, and their morphological and physiological characteristics have been determined. Table 2 summarizes the spore morphology formed by potent strains (MN2, MN39, and MN40), and their pigmentation ability in liquid media. In addition, the color of pigments produced by both aerial and substrate mycelium is illustrated. The results of microscopic studies of spore shape, and growth characteristics indicated that the morphological similarity among the 3 potent isolates was poor.

**Table 1:** The potential of 14 actinomycetes to produce antibacterial agents which are active against some MDR pathogens using cross-streak study

**samples**	**MDR Pathogens**					
	**Gram negative**				**Gram positive**	
	*E. coli*	*S. typhi*	*P. aeruginosa*	*A.baumannii*	*E. faecium*	*S. aureus*
MN1	+	-	+	+	-	+
MN2	+	+	+	+	+	+
MN6	-	+	-	-	+	+
MN8	+	-	+	-	+	+
MN13	-	-	+	+	+	+
MN17	+	-	-	-	+	+
MN19	-	-	+	+	-	+
MN23	-	+	+	-	+	-
MN27	-	-	+	+	-	+
MN30	+	-	-	-	+	-
MN38	-	-	+	+	+	+
MN39	+	+	+	+	+	+
MN40	+	+	+	+	+	+
MN44	+	-	+	-	+	+
Gentamicin	+	+	+	+	+	+
Ampicillin	+	+	+	+	-	+
Tetracycline	+	+	-	+	+	+
Cefazolin	+	+	+	+	+	+
Penicillin	+	+	+	+	+	-

(+): the growth of MDR pathogens has been inhibited; (-): no growth inhibition observed.

**Table 2:** Morphological and physiological characteristics of three potent actinomycetes on SCA medium

**characteristics**	**Isolates**		
	**MN2**	**MN39**	**MN40**
Spore-bearing hyphae	Simple (R)	Simple (RA)	Simple (R)
Pigmentation (liquid media)	Yellow	Brown	Dark Brown
The color of aerial mycelia	Gray	White	Gray
The color of substrate mycelia (Reverse color)	Yellow-Brown-Green	Yellow-Brown- Red	Yellow-Brown
R: straight- rectus; RA: retinaculum-apertum;- : negative; +: positive.			

R: straight- rectus; RA: retinaculum-apertum;- : negative; +: positive.

**Fig. 3:**
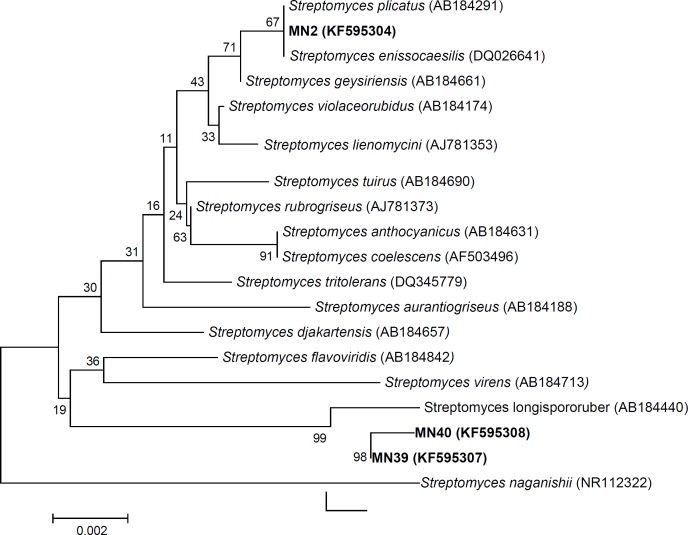
Neighbor-joining phylogenetic tree based on 16S rRNA gene sequence of potent strains. There seem to be relationships between selected strains and related species of the genus *Streptomyces*

**Fig. 4:**
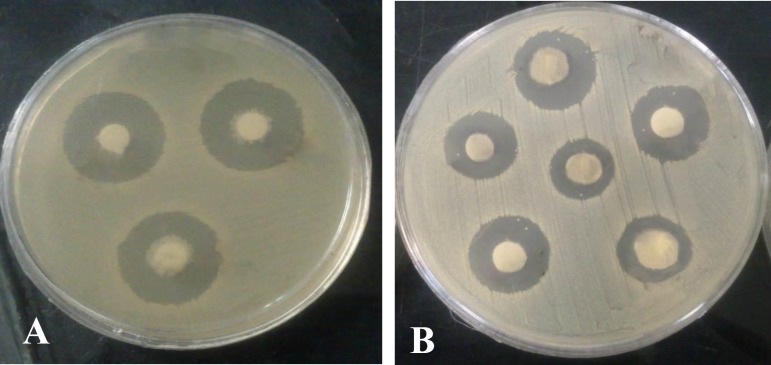
Antibacterial activities of the crude extract of isolates using disc diffusion method. A: antibacterial activity against *P. aeruginosa;* B: antibacterial activity against MRSA

**Molecular phylogeny of the selected strains**

The partial 16S rDNA sequences of the producer strains were determined, and a phylogenetic tree was constructed (Figure 3). Comparison of ribosomal DNA gene sequence revealed a strong relationship between isolated strains, and members of genus *Streptomyces* *sp*. The 16S rRNA gene sequence of MN2, MN40, and MN39 were submitted to GenBank under accession number of KF595304, KF595308, and KF595307, respectively. Strain MN2 was posed in the same branch with *Streptomyces plicatus *(AB184291), and* Streptomyces enissocaesilis *(DQ026641) shared 99.7% sequence similarity*,* while *Streptomyces geysiriensis *(AB184661) was found to be its neighbor strain. The 16S rRNA gene sequences of both MN40 and MN39 showed 99.7% similarity to *Streptomyces enissocaesilis*.

**Sub-merged fermentation**

The anti-MDR activities of the crude extracts obtained from the liquid culture of 3 selected isolates were evaluated using disc diffusion method (Figure 4).

The obtained-crude extracts inhibited the growth of MRSA and VRE more than other indicators which results in forming widest inhibition zone around the impregnated discs (Table3).

**Table 3:** Antibacterial activity of the potential actinomycete isolates using Kirby–Bauer disk diffusion method

**Test Strain**	**Zone of growth inhibition (mm)**		
	MN2	MN39	MN40
*E. coli* 1	13.0±1.4	22.5±0.7	14.0±1.4
*P. aeruginosa* 2	11.0±0.7	18.0±1.4	12.5±0.7
MRSA^3^	21.5±0.7	28.5±0.7	15.5±0.7
VRE^4^	25.0±0.5	24.5±1.4	13.0±0.7
*S. typhi* 5	14.0±1.4	16.0±1.4	18.0±1.4
*A.baumannii* 6	13.0±0.4	24.0±1.4	13.0±1.4

1 Escherichia coli;

2 Pseudomonas aeruginosa;

3 Methicillin-resistant Staphylococcus aureus;

4 Vancomycin-resistant Enterococcus (faecium);

5 Salmonella typhi;

6 Acinetobacter baumannii.

**Table 4:** Bioactivity of the selected *Actinobacteria* against *Vibrio* pathogens and their potential to produce some extracellular enzymes

***Strains***	***Enzymatic activities***		***Cross streak assay***		
	*Amylolytic*	*Proteolytic*	*V.harveyi*	*V. proteolyticus*	*V.parahaemolyticus*
*MN2*	*++*	*+++*	*+++*	*+*	*-*
*MN39*	*+*	*++*	*++*	*+++*	*++*
*MN40*	*+++*	*++*	*+++*	*++*	*+++*

Inhibition: +++ good; ++ medium; + slight; − nil.

The MN39 strain extract exhibited a remarkable activity against all indicator bacteria, whereas the growth inhibitory effect of MN40 crude extract against MRSA and VRE was low (Table 3).

**Exoenzyme and anti-vibrio** **activity **

Antagonistic activity of potent isolates has been evaluated against three species of *Vibrio*. Although bioactive metabolites produced by these three actinomycetes inhibited the growth of *V. harveyi* and *V. proteolyticus*, but MN2 isolate could not produce any biomolecule which could prevent the growing of *V. parahaemolyticus* (Table 4). In addition, all three actinomycetes produced extracellular enzymes including amylase and protease. However, the production of amylase by MN39 seemed to be lower.

## Discussion

Currently, one of the main threats to the healthcare in the world is the antimicrobial resistance, as stated by WHO (19). To combat this problem, there is an urgent need for probing new drugs with activity against antibiotic-resistant microorganisms. Natural compounds isolated from marine sources may play an important role to lead to the discovery of new antimicrobial agents. Due to the special condition of marine environment, actinomycetes are particularly desirable due to their high ability of producing bioactive compounds(8, 9).

The potent actinomycete strains isolated from the sediment of Caspian Sea have been identified as *Streptomyces* using 16S rRNA sequencing followed by BLAST analyzing. Identification via genetic approaches with a good speed is widely developed. Among molecular techniques used, 16S rRNA sequencing is a “gold standard” option due to the possessing the proper gene size, and availability of a large number of sequences in databases for comparison (20). The isolated actinomycetes showed antibacterial activity against some MDR pathogens such as MRSA and VRE. Sujatha et al. observed that marine *Streptomyces* species possess efficient antagonistic activity against MRSA (21) while Mercy Rajan and Kannabiran reported the ability of some marine *Streptomyces* isolates to produce antibacterial agents active against VRE (22). Sibanda et al. reported that crude extracts from the liquid culture of marine actinomycetes inhibited the Gram-negative bacteria more than Gram-positive bacteria strains (23). Conversly, the antibacterial activity of the strains isolated in the present study was higher against Gram-positive MDR pathogens. Bioactivity against MRSA and VRE is valuable due to many health problems raised from MRSA and VRE (24).

Extraction of bioactive compounds by ethyl acetate seems good enough because of observing a proper growth inhibition of indicators when disc diffusion method has been applied. Ethyl acetate can dissolve biomolecules with moderate polarity which can be used as drug in human body. Additionaly, this solvent is not miscible with water, which results in easy separation of organic from water phase (11, 12).

Being a probiotic in an aquaculture may arise from the capability of having antagonistic activity against aqua-pathogens such as *Vibrio* species (8) or producing extracellular enzymes which help in digesting the nutrients, leading to a better growth of host (9, 25). You et al. characterized some marine streptomycetes with anti-vibrio activity, and suggested their potential as probiotics (26). In 2013, the biomass production of 10 *Streptomyces* with starch-hydrolyzing activity that can be used as probiotics has been studied by Srisamai et al. (27). In the present study, the anti-vibrio activity of selected actinomycetes besides their ability to produce amylase and protease enzymes have been demonstrated. These findings show the potential of these strains to be adopted as valuable biocontrols, and aiding-growth agents in aquaculture industries. Although, it should be emphasized that for *Streptomyces* to be considered as a probiotic and/or a growth-stimulator factor in aquaculture ecosystems, performing further extensive studies are necessary.
